# Co‐Producing a Patient Reported Experience Measure (PREM) With and for People With Intellectual Disability

**DOI:** 10.1111/hex.70562

**Published:** 2026-01-23

**Authors:** Bronwyn Newman, Ling Wu, Laurel Mimmo, Beth Catlett, Matthew Van Hoeke, Maya Tokutake, Karen Phillips, Deborah Van Hoeke, Kim Bowen, Dalal Dawood Baumgarter, Pandora Patterson, Elizabeth Manias, Tracey Szanto, Nadine A. Hackl, Dhruve Basur, Mingye Li, Corey Adams, Patrick Olivier, Iva Strnadová, Julian N. Trollor, Reema Harrison

**Affiliations:** ^1^ Centre for Health Systems and Safety Research, Australian Institute of Health Innovation Macquarie University Sydney Australia; ^2^ Monash Action Lab Monash University Melbourne Australia; ^3^ The Sydney Children's Hospital Network Nursing Research Unit Sydney Australia; ^4^ Consumer Leadership Group, Australian Institute of Health Innovation Macquarie University Sydney Australia; ^5^ Fair Foundations Sydney Australia; ^6^ Faculty of Medicine, Nursing and Health Sciences Monash University Melbourne Australia; ^7^ Agency for Clinical Innovation NSW Health Sydney Australia; ^8^ Bureau of Health Information NSW Health Sydney Australia; ^9^ Faculty of Arts, Design and Architecture UNSW, Disability Innovation Institute (DIIU) School of Education Sydney Australia; ^10^ National Centre of Excellence in Intellectual Disability Health UNSW Medicine & Health, UNSW Sydney Australia

**Keywords:** accessible information, equity, inclusive research, intellectual disability, patient reported measures

## Abstract

**Background:**

Patient reported experience measures (PREMs) are widely used as key indicators of value in healthcare towards improved services but are rarely applied among people with intellectual disability. Incorporating the experiences of people with intellectual disability in PREMs data is vital as this group often encounter poor healthcare access and outcomes. This study reports the coproduction of accessible PREMs for people with intellectual disability from a 3‐year inclusive research project called Listen to Me.

**Objective:**

To coproduce a PREM suitable for people with intellectual disability.

**Method:**

Co‐production occurred through inclusive data collection methods in five hybrid co‐production workshops within an inclusive research project structure. Preliminary user testing of the Listen to Me PREM was conducted via semi‐structured interviews using the ‘think aloud’ method.

**Results:**

Co‐production included two people with intellectual disability, six people who support family members with intellectual disability, three researchers and three people with experience health service management or direct care. Preliminary user testing was completed by 11 people with intellectual disability with a range of communication needs and preferences. The resulting 9‐item Listen to Me PREMs are digitally‐enabled tools that include accessible features, such as large font and audible options to enable completion directly by people with a range of communication approaches.

**Conclusion:**

The Listen to Me PREMs provide an innovative tool to capture patent‐reported experiences directly from people with intellectual disability. By applying tools such as the Listen to Me PREMs, health services are better equipped to identify opportunities for improvement, to enhance access, quality and outcomes in health care delivery for consumers with high healthcare needs.

**Patient or Public Contribution:**

People with diverse abilities and communication preferences have been engaged both as members of the Listen to Me research team, and in all elements of data collection. The Listen to Me project has grown from a collaboration with consumers and all elements of this research engage with a diverse consumer group. The CanEngage Consumer Leadership Group (CLG) is central to all research activities including governance, data collection, analysis, preparation and dissemination of findings. The CLG has eight members, two members have intellectual disability and six are parents or siblings who support family members with intellectual disability to access health care. The CLG were involved in the design of the research proposal, reviewing and contributing to the ethics protocols, the coproduction of the Patient reported experience measure (PREM) and as authors of this paper. People who support family members with intellectual disability to access healthcare were involved in constructing and editing the paper. Two people with intellectual disability were involved in reviewing the accessible summary of the paper and provided feedback on clarity. Some authors are family members of people living with an intellectual disability. These family members are crucial advocates for people with a severe intellectual disability who do not have the capacity or capability of communicating using speech or writing.

## 1 Introduction

Patient reported experience measures (PREMs) are designed to collect and report on key indicators of value in healthcare, yet rarely incorporate the views and experiences of people with intellectual disability [1]. This is significant as people with intellectual disability are a group who have persistently experienced poorer access and quality of healthcare internationally [2, 3, 4, 5, 6]. PREMs are recognised as a key strategy to inform service planning and improvement implemented in health services worldwide [7]. Inclusion of people with intellectual disability in systemwide data collection via PREMs is a vital strategy to improve value in healthcare delivery and outcomes for this group with high healthcare needs.

Intellectual disability begins in the developmental period and is ‘characterised by intellectual difficulties as well as difficulties in conceptual, social and practical areas of living’ [8] and impacts approximately 500,000 Australians and 108 million people worldwide [9]. For people with intellectual disability, appropriate healthcare is often hard to find and when care is accessed it is often difficult to navigate, frequently insufficient or unsuitable [6, 10]. People with intellectual disability are a group who use healthcare frequently yet continue to experience some of the poorest care outcomes, representing low value care for consumers and health systems [11]. Healthcare quality often remains problematic, people with intellectual disability persistently experience poorer safety outcomes, more reported incidents and more frequent readmission for chronic or persistent conditions than the average patient population [6, 12]. Frequent readmissions have been identified across the lifespan and are often attributed to failures in the management of and or recognition of chronic or persistent conditions representing poor value to both consumers and health systems [3, 13, 14]. These systemic inadequacies have been recognised internationally as directly contributing to people with intellectual disability experiencing poorer health outcomes than their peers in the wider community [2, 4, 5, 15, 16]. Considering these well‐documented disparities, it is vital that people with intellectual disability are included in opportunities to lead, plan and inform care, both at the individual and health service levels to ensure healthcare that delivers value to patients, families and the wider system [7, 17].

Routinely used PREMs often include complex written information, large numbers of response items and are provided in formats that may prove difficult to access for people with intellectual disability [18]. For example, the number of questions, complicated wording, small check boxes and lack of images can make PREMs difficult to navigate and understand [7, 19]. PREMs are used in condition or service specific settings, as well as across health service systems, utilising tools such as the National Health Service (NHS) Picker survey in the United Kingdom, Consumer Assessment of Healthcare Providers and Systems (CAHPS) in the United States, the Nordic Patient Experiences Questionnaire (NORPEQ), or the Australian Hospital Patient Experience Question Set (AHPEQS) in Australia [17, 20]. PREMs are often collected via questionnaire, or interview to inform healthcare service planning or enhancement after the completion of a hospital stay or appointment [21]. There is a significant association between patient experience of hospital care and negative health outcomes [22, 23]. Technological advances provide a range of opportunities for enhanced accessibility that are yet to be explored and applied in PREM tools. There is a recognition of the need for accessible PREMs suitable for people with intellectual disability internationally, evident in the development of a tool for use in a UK paediatric setting [24]. Yet there remains an absence of validated tools that can be completed directly by adults, young people and children with intellectual disability, or that have been designed with this population to address their needs [24].

PREMs accessible for people with intellectual disability can provide an opportunity for better understanding of need and guide tailored service provision to enable better value, high quality care. A recent (2022) literature review demonstrated that internationally, no validated PREMs suited to system‐wide implementation has been developed for people with intellectual disability to report their own experiences. This finding illuminated the need for the adaptation or development of suitable measures [19]. Engaging with people with intellectual disability in the design of suitable PREMs to address longstanding disparities in healthcare access and outcomes is vital [16, 25, 26]. The absence of PREMs suitable for people with intellectual disability to report their healthcare experiences alongside wider patient reporting to be included in existing data sets has led to the development of the Listen to Me project. Details of the overall project are reported elsewhere (under review). Named by the consumer leads, Listen to Me is a 3‐year inclusive research project which involves people with intellectual disability in all elements of research, encompassing governance, data collection, and as participants [27]. The aim of this study was to co‐produce a digital PREM suitable for people with intellectual disability that reflects what is important to consumers and is actionable for healthcare professionals. In this paper we describe the co‐production approach, including preliminary testing and introduce the output: the Listen to Me PREMs 1.0. The co‐production approach involved working with people with intellectual disability, their family members and supporters through collaborative research methods that prioritise the involvement of people with intellectual disability at all stages of the research process. Coproduction included engaging all consumers in conceptualisation, research question formulation, research design, research conduction, evaluation, testing and reflection [27, 28, 29, 30]. This approach is rooted in the principles of inclusive research, which emphasises the importance of involving people with intellectual disability, family members and supporters in the research process to ensure that their experiences, perspectives, and needs are reflected in depth in the research [25, 31]. An Easy English summary of this research is available (Supporting Information File 1).

## 2 Methods

In the absence of a preferred reporting framework for co‐production, the process is reported to align with the Problem, Objective, Design, (end)‐Users, Co‐creators, Evaluation and Scalability (PRODUCES) framework, developed to enable a systematic reporting of co‐creation activities [32].

### 2.1 Objective

To co‐produce a PREM with people with intellectual disabilities and the people who support them suitable for patient experience data collection in Australian healthcare settings.

### 2.2 Ethics

Ethics approval was gained from Human Research ethics Committee at Macquarie University, reference number: 520241735259588.

### 2.3 Co‐Production Methodology

Co‐production is defined as ‘a process of collaboration and collective decision‐making, which involves changing the relations of research traditionally separating users and producers’ [27]. Our approach employed a multi‐methods design to facilitate creation of an accessible PREM. Creating an environment suitable for coproduction with people with intellectual disability required the incorporation of inclusive research strategies throughout the project in all communication, governance and data collection activities [27, 33]. All research was scaffolded by an inclusive governance structure characterised by consumer engagement in all activities, centred around a Consumer Leadership Group (CLG). The CLG comprised of eight people, two with lived experience of intellectual disability, four parents of children and young people with intellectual disability and two siblings with experience supporting an adult family member with intellectual disability to access health services [34]. Consumer co‐researchers were renumerated for attendance, travel, workshop preparation and research tasks, including reviews related to preparation of this manuscript. All project communication was provided in Easy Read formats [27, 33, 35, 36, 37, 38].

### 2.4 End Users and Co‐Creators

**Figure 1:**
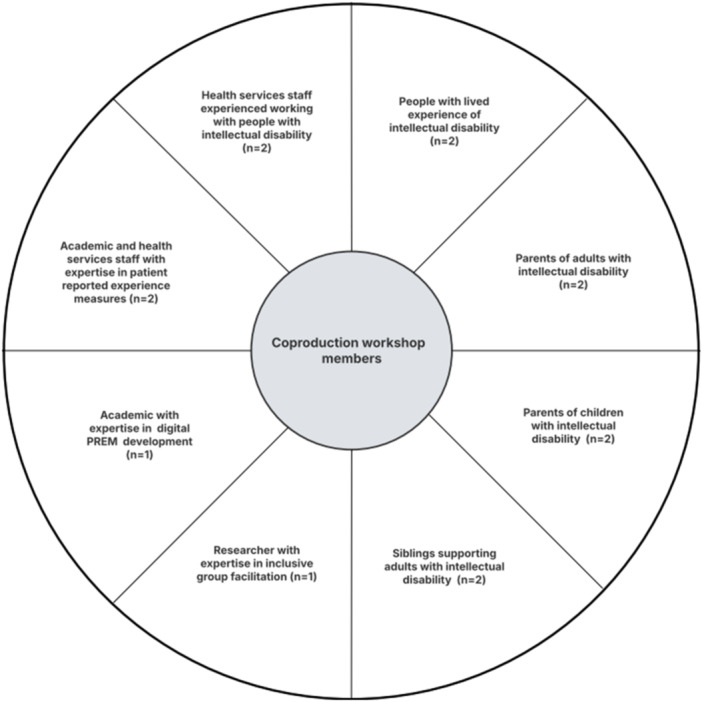
Co‐production workshop members.

End users for the Listen to Me PREM include people with intellectual disability aged 6 years and older who will complete the PREM, their supporters, hospital staff or others who may support completion, and health system staff who will use the resulting data for quality improvement. Co‐production members represented each of these user groups, summarise in Figure 1.

Co‐production group members were drawn from the Listen to Me project team to provide a breadth of professional and lived experience expertise. Multidisciplinary academic team members and hospital staff with expertise in health survey development, digital design, clinical expertise, intellectual disability health and co‐production facilitation contributed to the process by attending sessions or providing asynchronous input to the design. Ongoing digital design, guidance and engineering was provided by project partner Monash Action Lab, Monash University throughout the coproduction. The lived experience of the co‐production group members guided the focus of the PREM and informed the CLG's choice of project name *Listen to Me*.

### 2.5 Procedure

**Figure 2:**
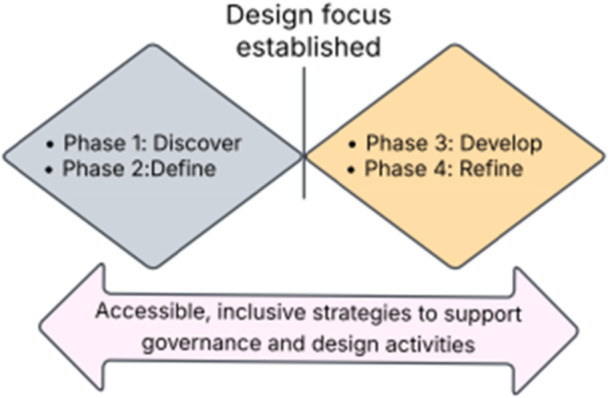
Co‐production design approach.

We used the ‘double diamond’ design model (Figure 2) to guide the co‐production process, by first ‘discovering’ and exploring current practice, defining the scope of our work together, and then focusing on development and delivery to refine the PREM. The Listen to Me co‐production workshop series was conducted with the CLG to design and produce the PREM in five hybrid workshops. Co‐production workshop methods sought to create an environment that enables ‘consumers to become equal partners in the improvement process for health services’ [39].

Pre‐workshop meetings enabled the team to develop trust, understand each other's communication styles, incorporate supports and tailor activities and key elements in creating an inclusive research environment. The CLG established that their preferred mode for workshops was through onsite meetings to suit members with lived experience of intellectual disability, and online attendance via a Zoom (Zoom Communications 2024) online platform available for all CLG members. A hybrid of in person and online meeting platforms were utilised (based at Macquarie University) with an asynchronous component to accommodate follow up, clarification, feedback and input between meetings. Onsite attendance offered the opportunity for CLG members to attend for preparation and activities in person prior to other participants joining online, and to stay on afterward for questions and clarification over lunch as appropriate meeting times were scheduled to ensure meeting times were suitable for CLG members with intellectual disability to attend onsite with their supporters and any other CLG members available to attend in person (see Table 1 for attendee numbers). Feedback and design preferences were provided to research partners, Monash Action Lab, specialised in digital PREM development to create and adapt the digital PREM.

**Table 1:** Co‐production workshop summary.

Design phase	Workshop	Aim	Activities	Outcome
*Phase 1: Discover*	Pre‐Workshop Meeting	To build trust and rapport with co‐production group and establish shared understanding of project scope.	body mapping [39] relationship building over morning tea and lunch.	Team members established: communication preferences workshops will be held in person with online attendees to join after initial in‐person preparation activities. Focus topics of remaining workshops.
	Workshop 1	To establish shared understanding of what a PREM is and how they are used in Australian hospitals.	Facilitated discussion using social stories. Discussed data collection and PREMs with NSW Health staff.	The group gained an understanding of: how PREM data is collected and used in NSW. what is most important for NSW health to use in service planning.
*Phase 2: Define*	Workshop 2	To prioritise topic areas to include in the PREM.	Accessible Voting activity: easy read style picture and text to represent key question topics from the literature (*n* = 13) to determine preferences.	Voting on topics to include, identified importance using a scale of Yes, No and Maybe. Between workshops researchers distilled results to summarise the most important topics. *(Figure 3)*
*Phase 3: Develop*	Workshop 3	To explore what types of questions we could ask.	Explored what worked well and not so well using existing surveys.	This workshop reinforced the benefit of: images flexibility in approaches to asking and answering questions Benefit of audio options
	Workshop 4	To experiment with ideas created by Monash Action Lab.	Hybrid meeting with printed and tablet versions of PREM 0.1 prototype with various question types for exploration.	Adaptations suggested to Action Lab team member (online) Scheduled additional meeting for user testing of the PREM with the group.
*Phase 4: Deliver and Refine*	Workshop 5	Testing the PREM prototype.	User testing with CLG members with lived experience of intellectual disability.	Further changes to develop PREM 0.2
	Preliminary User testing	User testing of the PREM beyond our Consumer Leadership Group.	Think‐aloud interviews with 11 people with intellectual disability	Changes incorporated to PREM *(Figure 4)*

We applied flexible, arts‐based methods in coproduction workshop activities familiar to CLG members with lived experience of intellectual disability from previous co‐researcher training [33, 35]. Methods included body mapping [40], accessible voting and craft‐based activity to explore issues and preferences, outlined in Table 1. Development and maintenance of relationships of trust was central to the coproduction process, informing and supporting various practical elements implemented throughout the research and grounded in relevant guides and inclusive research principles [27]. Practical strategies were informed by inclusive research principles and guidelines [36, 37, 38], developed iteratively, and tailored to suit participants. To ensure all participants had opportunity to express their views contact with researchers between meetings was encouraged, and researchers regularly checked in with team members with intellectual disability and their supporters. Table 1 presents a summary of methods and outcomes for each coproduction activity, including the workshop series.

Workshop content was developed to align with the double diamond design principles throughout the co‐production process (Figure 2) [41]. Workshop 1 involved planning with the group to devise a suitable approach, the double diamond design principles framed these discussions and guided the work to follow. The principles informed co‐production, with research aims and activities conducted in these four phases outlined in Table 1.

**Figure 3:**
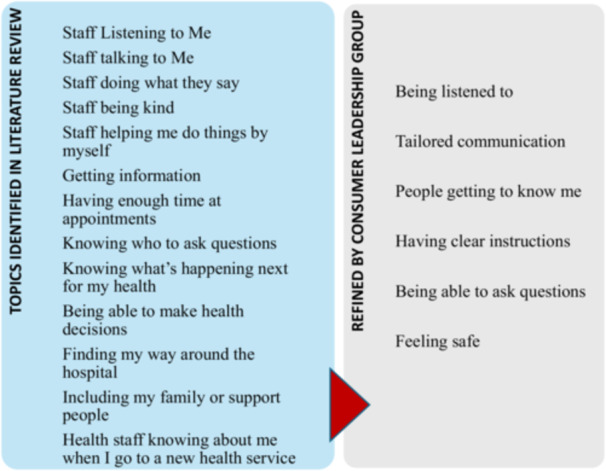
Refining topics to include in the PREM*.*

**Figure 4:**
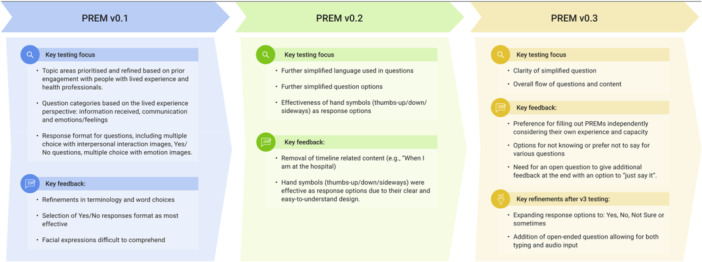
PREM prototype development*.*

The topic areas to be included in the PREM were explored in Workshops 1–3 and the key topic areas prioritised for inclusion remained consistent throughout the development of the PREM (Figure 3). Through the workshops, the PREM developed via 3 main iterations and a summary of key changes in each iteration are listed in Figure 4. An initial prototype of the Listen to Me PREM 0.1 was built after workshop 3, elements were tested, then refined by Monash Action Lab following subsequent workshops to create. Opportunities for further input about PREM development were available in between workshops as needed, and several smaller online meetings were conducted with members of the group to clarify their preferences. Changes suggested by the group primarily related to terminology and word choice. After gaining insights from the co‐production workshop process, Monash action Lab developed the Listen to Me 0.2 to evaluate through preliminary user testing. Listen to Me v0.3 was then confirmed by the coproduction group after evaluation via preliminary testing.

### 2.6 Evaluation

We conducted user‐testing of the Listen to Me PREM 0.2 to understand how people with a diverse array of needs and communication preferences beyond those within the co‐production group interact with the PREM, and canvass suggested adaptations to further refine the PREM content, usability and accessibility. For the purpose of user testing, the Listen to Me 0.2 prototype was developed on a third‐party software (Protopie‐Studio XID 2025) on designated tablets. Think‐aloud interviews [42] were used to explore the experiences of 11 people with intellectual disability with varied communication preferences and support needs using the PREM prototype. A summary of participant demographic information and interview length is available in Supporting Information File 3: User testing interview participant summary.

Participants were recruited via project partner agencies who worked with people with intellectual disability. The invitation was sent via email invitation, promoted in regularly conducted group meetings or forums, and accompanied by a verbal explanation of project processes when required. Easy Read information about the Listen to Me project was made available to participants, and they were invited to take part in an interview with the researcher at a time and location convenient to the participant. Project information was provided electronically prior to interviews, and a hard copy was used to facilitate discussion and confirm consent at the time of interview. All participants were able to consent independently, one chose to provide verbal consent only and others signed the easy read form (see Supporting Information File 3 for detail). Think‐aloud interviews were the chosen method as this approach provides an opportunity for observation and exploration of specific interactions of people with diverse needs with both the technology and concepts. Direct questions were included to elicit feedback about specific elements of the PREM to further refine the prototype (Supporting Information File 2).

A researcher (B.N.) attended 10 interviews in person and one online with support, interviews ranged from 8 to 32 min and participants were from a variety of geographic locations with diverse support needs (Supporting Information File 3). All interviews were audio recorded. Two participants chose to participate with a support person present, both accessing support to ensure understanding of consent and research processes, while the online participant also received technological support to attend the interview. All participants were reimbursed to acknowledge their expertise and time commitment at the time of the interview.

Initial identification of feedback themes was conducted by B.N. and further explored with CI (R.H.) and prototype developer (L.W.). Feedback from interviews was directly related to aspects of the PREM's usability. For example, when discussing commentary about using thumbs up images to indicate choice Consumer Participant ID 1 commented' and thumbs down, they understand. In the situation of talking to most people with disabilities, they understand'. Similarly, when considering the option of face emojis Consumer Participant ID 3 explained ‘So the happy one [emoji] is… yeah very happy smiling but okay is yeah so‐so kind of thing and what all those I don't know those two uh these two kind of are like they are the same’.

Researcher (B.N.) presented annotated findings from transcribed recordings and field notes to develop key themes in consultation with research team members (L.W., L.M.). Key findings were grouped according to themes of ‘understandability, clarity of choices, look and feel, and usability’ using an Excel spreadsheet in order of PREM questions to inform refinements. A key recommendation from preliminary testing and discussion with pilot sites was the adaptation to include inpatient experience in the PREM. This is reflected in the Listen to Me PREMs 1.0 and 2.0 developed.

To enable recommendations for further refinement of the PREM to 0.2, a fifth workshop was conducted with the co‐production group to review and test the functionality of the Listen to Me 0.3 after all suggested adaptations had been made. The primary focus of this workshop was to observe how CLG consumers interacted with the prototype on an iPad, and the enablers and barriers to completion. Prototype developer (L.W.) conducted interviews with CLG members. The feedback reflected that the consumers were able to enable to complete the prototype independently. Further minor changes to images and font placements were confirmed, and feedback regarding the need for flexibility to incorporate outpatient care was incorporated, leading to two PREMs: the Listen to Me Inpatient PREM 1.0 and the Listen to Me Outpatient PREM 1.0. The full development process and iterative changes are summarised in Figure 4.

### 2.7 Scalability

Further steps to enable scaling of the Listen to Me PREMs include a Delphi study and pilot study across a range of Australian hospitals. First, given the diverse communication needs and preferences of the target population, an accessible Delphi study will be conducted to ensure the PREMs items have relevance and clarity for the wider community. These findings will inform further refinement. Piloting the PREMs to capture the experiences of people with intellectual disability in Australian hospitals and outpatient clinics will be further used to explore its feasibility for implementation. Pilot will include exploration of support needs of hospital staff and consumers, costs associated with PREM use, and generating data that can be used to determine the PREMs reliability and validity. Findings will be used to inform capability development to translate the Listen to Me PREMs into healthcare settings across Australia. Findings from these studies will be reported when complete.

### 2.8 Output

We produced the Listen to Me inpatient and outpatient PREMs 1.0 using the co‐production approach outlined (Table 1).

The co‐produced PREMs are suitable for use via touch screen device or mouse. The survey questions appear with large black font, and images accompany questions. All questions are available as text to read, or via audio. Participants can input responses by selecting a visual option on the screen, for the final open question, responses can be typed or recorded and a draft user guide has been developed (Supporting Information File 4). For research purposes, six additional demographic questions were incorporated into the PREMs 1.0 to facilitate completion and data analysis. These can be adapted for use in varied settings as demographic information may not be required if the PREM is integrated for health service use. Images of the PREM questions are provided (Supporting Information File 5).

The resulting Listen to Me PREM version 1.0 is available as a 9‐item inpatient and Listen to Me outpatient tool. Both comprise of eight closed items and one open item with embedded scoring to facilitate validation and analysis. With a score of 0, 5 and 10 allocated to the response options of ‘no, sometimes and yes’ (Table 2). The outpatient version uses the term ‘clinic’ rather than ‘hospital’ relevant to the setting. All Listen to Me resources and related survey tools are available on the Listen to Me Webpage https://www.mq.edu.au/faculty‐of‐medicine‐health‐and‐human‐sciences/departments‐and‐schools/australian‐institute‐of‐health‐innovation/our‐research‐centres/centre‐for‐health‐systems‐and‐safety‐research/our‐research/listen‐to‐me.

**Table 2:** Listen to Me PREM1.0 items with scoring.

	Response options and scoring		
	No	Sometimes	Yes
Inpatient items			
1. Did the people who cared for you at the hospital get to know you?	0	5	10
3. Did the people who care for you at the hospital find out how you like to communicate?	0	5	10
4. Did the people who work at the hospital listen to you?	0	5	10
5. Did the people at the hospital give you clear instructions?	0	5	10
6. Did people who work at the hospital tell you things in a way that you understand?	0	5	10
7. Did people at the hospital let you ask questions?	0	5	10
8. Did you feel safe when you are staying in hospital?	0	5	10
9. Would you recommend this hospital to a friend?	0	5	10
10. Is there anything else you would like to tell us?	*(Free text entry)*		
Outpatient items			
1. Did the people who cared for you at the clinic get to know you?	0	5	10
2. Did the people who care for you at the clinic find out how you like to communicate?	0	5	10
3. Did the people who work at the clinic listen to you?	0	5	10
4. Did the people at the clinic give you clear instructions?	0	5	10
5. Did people who work at the clinic tell you things in a way that you understand?	0	5	10
6. Did people at the clinic let you ask questions?	0	5	10
7. Did you feel safe when you are at the clinic?	0	5	10
8. Would you recommend this clinic to a friend?	0	5	10
9. Is there anything else you would like to tell us?	*(Free text entry)*		

## 3 Discussion

We co‐produced the 9‐item Listen to Me Inpatient and Outpatient PREMs 1.0 with and for people with intellectual disability, collaborating with families, supporters, healthcare practitioners and healthcare staff engaged in statewide data collection. People with intellectual disability had central roles in all elements of coproduction, from project inception through to coproduction workshops, preliminary PREM testing, and in project governance. Significantly, people with intellectual disability named the project and provided practical suggestions about the PREM's digital functionality and accessibility. The novelty of the Listen to Me PREM is not only that it was created and refined by a diverse group of people with intellectual disability and their supporters, but that it is designed to be implemented across healthcare and benchmarked with wider patient experience data. Enabling collection and integration of experience data from people with intellectual disability in routine PREM collection can inform more targeted service and resource planning, enabling higher value care for services and consumers. The varied roles and interests of the Listen to Me project team enables ongoing partnerships with varied health agencies as facilitators of PREM use, and end‐ users of PREM data. The diversity of the Listen to Me partner members provided ready access to expertise throughout coproduction, which will continue during the ongoing implementation and evaluation of this work.

International recognition of the need for reform to enable the provision of equitable, value‐based healthcare emphasises the central role of people with intellectual disability to inform quality care and drive reform [11, 43, 44]. Hearing directly from people with intellectual disability is vital, as the association between patient experience and poor outcomes, and the value of using patient experience to enhance healthcare quality is well‐established [22, 23, 45]. The Listen to Me PREMs are designed to capture patient experiences in an accessible way for independent completion by many people with intellectual disability and provide this data to health agencies in an accessible format. As such, Listen to Me PREMs can enable the experiences of people with intellectual disability to be captured in service‐wide data sets to inform service planning and care alongside general population data to enable need to be more effectively identified and addressed.

The Listen to Me PREMs are novel as they were purposefully and collaboratively designed to enable the experiences and priorities of people with intellectual disability to inform improved healthcare delivery, outcomes and access. The opportunities afforded by accessible surveys to enable communication of views, experiences and preferences have been shown to be effective and meaningful to consumers and service providers [1, 18, 46]. While bespoke surveys have been demonstrated to offer value to specific disability service or care planning, the translation of the experience data of people with intellectual disability to inform wider service planning and reform was less evident in the literature [1, 24]. Including the experience data from people with intellectual disability to be included in datasets which inform service planning facilitates more targeted planning aligned with principles of value‐based care, central to Australian Healthcare [47]. The Listen to Me PREMs build upon the existing evidence base informing how to create accessible data collection tools in the coproduction of an accessible, validated PREMs to implement across a variety of health service types to enable tailored service design informed by what matters to consumers.

Working together to co‐produce the Listen to Me digital PREMs reinforced findings in related literature, underlining the importance of fostering trusting relationships between all team members to enable open discussion, activity tailoring and clear communication [48, 49]. Inclusive researchers advocate for the use of flexible approaches alongside various activity‐based methods in well‐evidenced guides and practice models [33, 36]. Listen to Me has further demonstrated the ways that inclusive strategies such as timing, meeting formats and tailored communication can be incorporated in all elements of health service research coproduction including governance, data collection and decision making. This approach enables research that captures and represents what matters to consumers whose views are often not captured by traditional methods in health research. Further detail and evaluation will be published.

### 3.1 Strengths and Limitations

A strength of the coproduction approach was the commitment and goodwill of co‐production members. Willingness to invest in long‐term working relationships with consumers as colleagues and cross sector engagement in both formal and informal opportunities to build relationships, offered a robust foundation for this research.

The use of inclusive, tailored methods in research governance and data collection was a significant strength of this research, and often an iterative, adaptive process. We encountered various challenges within the project, such as variable availability for face‐to‐face meetings, differing communication preferences and diverse learning preferences. However, incorporating an inclusive tailored approach was useful and will help with undertaking future health services research. Challenges related to diverse elements such as creating an inclusive hybrid meeting model, developing accessible engagement opportunities in tasks which did not readily lend themselves to activity or art‐based approaches, negotiating employment contracts, changes in timing and shifts in plans. Providing opportunities to follow up after workshops and continue discussions online were valuable strategies to ensure all members of the team had opportunity to consider their views and contribute to the project processes. Established relationships and clear channels of communication were helpful in negotiating a way through these issues as they arose. An evaluation and discussion of the coproduction approach, including governance is reported elsewhere with further detail of the strategies implemented to enable inclusion.

We recognise the limitations of co‐producing the Listen to Me PREMs to be suitable for a diverse group with a relatively small co‐production team in an Australian setting. Engaging purposefully with members of the community with intellectual disability, their families and supporters, for the duration of the PREM coproduction provided rich insights into what is important for health consumers. These insights informed the PREMs development and enabled targeted and tailored testing of the PREM. Embedding the coproduction in a large well supported project with motivated partners enables greater opportunity for exploring the appropriateness of the Listen to Me PREM. Further exploration and refinement of the PREMs will be undertaken by piloting the PREM in diverse settings and conducting an inclusive Delphi study (underway). Once validated in an Australian context testing beyond the Australian health system could determine adaptations required for international use. We recognise that there are potential adaptations and uses of this cognitively accessible tool among various populations in diverse settings that would be valuable to explore in future research.

## 4 Conclusion

The Listen to Me digital PREMs are the first PREMs designed for health wide system use, co‐produced with and for people with intellectual disability. Capturing the experiences of people with intellectual disability, a group known to be high users of healthcare and experience poorer outcomes than their peers in the community, is vital to inform value‐based planning and service. When creating the Listen to Me PREM to inform change, collaboration with all stakeholders throughout the process was essential. Including perspectives of consumers, alongside practitioners and policy makers, enabled the creation of a PREM that is: accessible to a diverse group of people with intellectual disability, can enable what matters to consumers to inform service improvement, and ensures data usability by health services. The data gathered via the Listen to Me digital PREM will enable health services to focus on significant areas for improvement as identified by people with intellectual disability, supporting improved service planning, outcomes and greater equity in healthcare.

## Author Contributions

**Bronwyn Newman:** conceptualisation, investigation, writing – original draft, methodology, writing – review and editing, formal analysis, project administration, data curation, resources. **Ling Wu:** conceptualisation, investigation, methodology, writing – review and editing, software, formal analysis, resources, data curation, visualisation. **Laurel Mimmo:** conceptualisation, investigation, writing – review and editing. **Beth Catlett:** investigation, writing – review and editing, project administration, resources, conceptualisation, methodology. **Matthew Van Hoeke:** conceptualisation, writing – review and editing, investigation, methodology, visualisation. **Maya Tokutake:** conceptualisation, writing – review and editing, resources, investigation, visualisation. **Karen Phillips:** conceptualisation, writing – review and editing, resources, investigation, visualisation. **Deborah Van Hoeke:** conceptualisation, writing – review and editing, resources, investigation, visualisation. **Kim Bowen:** conceptualisation, writing – review and editing, resources, investigation, visualisation. **Dalal Dawood Baumgarter:** conceptualisation, writing – review and editing, resources, investigation, methodology, visualisation. **Pandora Patterson:** conceptualisation, writing – review and editing, resources, investigation, visualisation. **Elizabeth Manias:** investigation, conceptualisation, writing – review and editing, methodology. **Tracey Szanto:** conceptualisation, writing – review and editing, methodology. **Nadine A. Hackl:** investigation, writing – review and editing. **Dhruve Basur:** software, writing – review and editing. **Mingye Li:** software, writing – review and editing. **Corey Adams:** writing – review and editing, resources, visualisation. **Patrick Olivier:** investigation, software, conceptualisation, writing – review and editing. **Iva Strnadová:** investigation, conceptualisation, writing – review and editing. **Julian N. Trollor:** conceptualisation, investigation, writing – review and editing. **Reema Harrison:** conceptualisation, investigation, funding acquisition, methodology, writing – review and editing, project administration, supervision, formal analysis.

## Ethics Statement

Ethics approval was gained from National Health and Medical Research Council Accredited Human Research Ethics Committee (HREC) at Macquarie University, reference number: 520241735259588.

## Conflicts of Interest

The authors declare no conflicts of interest.

## Supporting information


**Supplementary File 1:** Listen to Me Easy English.

**Supplementary File 2:** Interview schedule.

**Supplementary File 3:** Interview participant information.

**Supplementary File 4:** Listento Me PREM User Guide.

**Supplementary File 5:** Listen to Me Hospital Prem Items.

## Data Availability

The data that support the findings of this study are available on request from the corresponding author. The data are not publicly available due to privacy or ethical restrictions.
