# Correction to “Co‐Producing a Patient Reported Experience Measure (PREM) With and for People With Intellectual Disability”

**DOI:** 10.1111/hex.70702

**Published:** 2026-05-21

B. Newman, L. Wu, L. Mimmo, et al., “Co‐Producing a Patient Reported Experience Measure (PREM) With and for People With Intellectual Disability,” *Health Expectations* 29 (2026): 1‐11, https://doi.org/10.1111/hex.70562.

The Author Dhruv Basur's first name has been updated from “Dhruve” to “Dhruv” and the online version of this article has been corrected accordingly.

We apologize for this error.

