# Supplementary material for: Co‐Producing a Patient Reported Experience Measure (PREM) With and for People With Intellectual Disability
Source: Health Expect. 2026 Jan 23;29(1):e70562. doi: 10.1111/hex.70562 (PMC12828785; doi:10.1111/hex.70562)
Supplement: Supplementary file 1 — Listen to Me Easy English. [file HEX-29-e70562-s002.pdf]

Easy English Version

Listen to Me: Making a way for people with intellectual disability to share about their health care

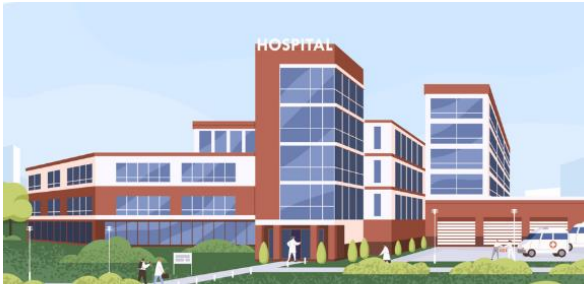

Why This Project Matters

We know that people with intellectual disability do not always get the care they need at the doctor or hospital.

We need a way of finding out what people with disability think about their health care. Then we can work together to make things better.

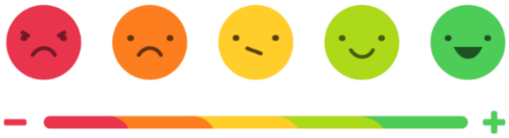

Doctors and nurses ask people what they think about their care. These questions are called Patient Reported Experience Measures or PREMs.

Hospitals collect everyone's answers to the PREM questions. The hospital leaders read them and find out how to make things better.

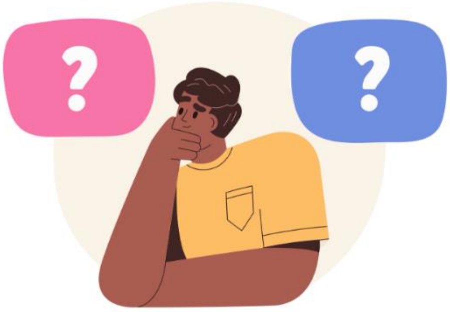

But the questions are difficult for some people. Some people with intellectual disability cannot answer the questions. Then the hospital leaders do not hear what people with intellectual disability think about their hospital.

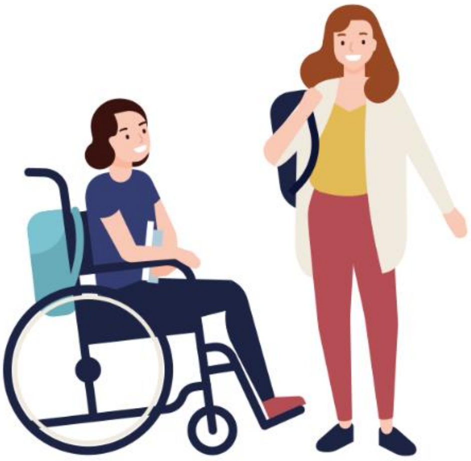

The research team at Macquarie University wanted to fix this. We started a project called Listen to Me. We are making a new, easy survey.

It will take **3 years** to make it.

This new survey will help people with disabilities to say what they think about their care in hospitals.

What We Did

Our team has lots of people who want to make health care better for people with intellectual disability.

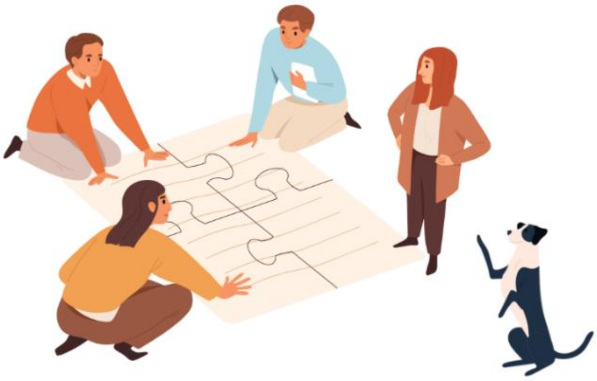

We worked together as a team to make the PREM.

11 people with intellectual disability as well as their families and researchers decided what questions to ask.

To make the PREM we used activities like drawing and sharing ideas.

We tried it out and listened to everyone's ideas to make the survey even better.

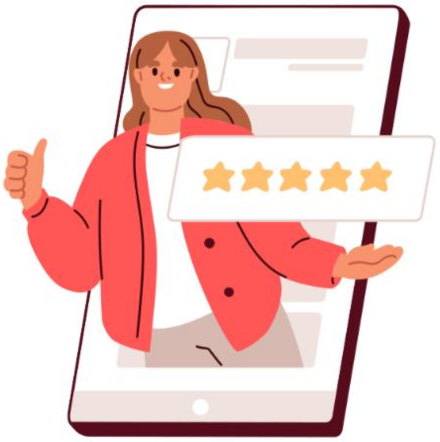

What We Made

A new survey called the Listen to Me PREM.

The Listen to Me PREM has 9 questions.

The Listen to Me PREM means that people with intellectual disability can answer the questions now. They can say how they feel about their care. The hospital leaders will know what they think could be better.

We are trying it out to see if it works. People with intellectual disability are helping us make the Listen to Me PREM. They are trying it out.

Why This is Important

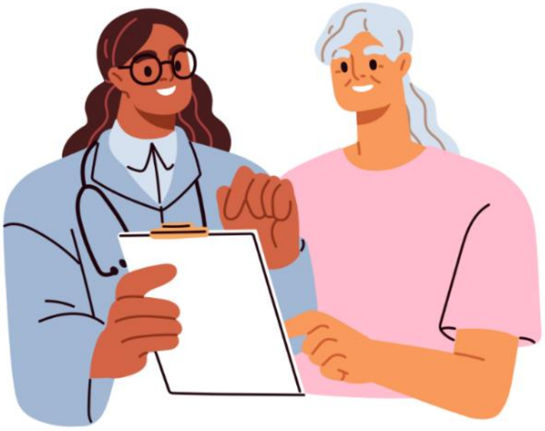

Everyone should have a way to say what they think about their health care.

The Listen to Me PREM can help people with intellectual disabilities say what they thought about being in hospital.

This helps hospital leaders listen, learn, and get better at caring for everyone.

This is important work.

Our team of people with intellectual disability and parents and researchers are doing something new and important.
