# Supplementary material for: Co‐Producing a Patient Reported Experience Measure (PREM) With and for People With Intellectual Disability
Source: Health Expect. 2026 Jan 23;29(1):e70562. doi: 10.1111/hex.70562 (PMC12828785; doi:10.1111/hex.70562)
Supplement: Supplementary file 2 — Interview schedule. [file HEX-29-e70562-s004.pdf]

SURVEYS prototype Think Aloud Interview Schedule

1. INTRODUCTION SCRIPT

- Explain what a patient reported experience measure (PREM) is – a survey for people to tell health services what they think about their care. The survey is completed on an iPad (provided)
- This is a study to help make a survey so that people with intellectual disability can do a survey about their time in hospital.
- I have some Easy Read information about what a Patient Reported Experience Measure is. Have you seen it? We can go through it if you have questions. (Appendix 12)

- If you decide that you do not want to take part anymore, please just let me know and we can stop. We can also stop for a little while if you need a break.

- If you are happy, we will record the interview, so we can listen again to what is being said. Everything we talk about here will be confidential. We will take care to make sure that all the information you share with us is kept safely and securely.

- Is there anything you would like to ask me?

- OK, so I will tell you a little more about what we will be doing today.

- We're interested in your views of the PREM survey we have been making. All you have to do is use the survey as you would if I was not here and say your thoughts out loud as you do it. To help you think aloud you may find it useful to read aloud or tell me what you are clicking on and why. You may find at times I will say aloud what you have clicked on or what part of the scorecard you are looking at – this is just so when we listen to your views again we know what question you are talking about.

- This is not a test. There are no right or wrong answers, so please say any thoughts, even if you think they might not be important.

- Please do feel free to say good and bad things about the survey as these will help us to improve it. Your views about the survey are important so the more you can tell us the better.

- I won't be able to answer your questions as we go through, but I can answer anything at the end. So if you ask me a question while you're looking at the survey, I will probably say that we can talk about it at the end.

- After you have finished looking at the survey, I would like to have a chat with you about your overall views of it.

- Ok so can I confirm you are happy to participate and start recording now?

2. **THINK ALOUD TOPICS AND PROMPTS**

- **First impressions: What do you think when you first see the survey?**
  - What do you think you need to do to get started?
  -

- **For each survey item:**
  - What do you think this question is about?
  - Do the pictures help you understand the question?
  - How do you choose the response options.
  - Are there enough options to choose from?
  - Is there anything that doesn’t make sense?
  - What would make it easier to understand?
  - Is there anything missing?

  - **Do you think that the question is clear?**

    4 point LIKERT SCALE WITH EMOJIS

    YES .....kind of ......not really...NO
  - **Do you think that the question is important to have in our survey?**

    4 point LIKERT SCALE WITH EMOJIS

    YES .....kind of ......not really...NO

3. **AFTER PARTICIPANT HAS COMPLETED THE PREM SURVEY :**

- Overall, what do you think about the survey?
- Can you tell me about anything you thought was particularly good about it?
- Can you tell me anything that we should change?
- Anything else you would like to tell us?

4. **THANK YOU & GOODBYES**

We are planning to make changes to the survey after we hear what people think. Would you be happy for us to contact you when we have made the changes so that we can try it out again?

If you are happy to have another try of the survey I will stop recording now and we will make sure we know the best phone number or email address to use to contact you. You don’t have to try it out again and even if you say yes now you can change your mind.

We appreciate you taking the time to tell us what you think. Voucher to say thanks.
