# Supplementary material for: Co‐Producing a Patient Reported Experience Measure (PREM) With and for People With Intellectual Disability
Source: Health Expect. 2026 Jan 23;29(1):e70562. doi: 10.1111/hex.70562 (PMC12828785; doi:10.1111/hex.70562)
Supplement: Supplementary file 3 — Interview participant information. [file HEX-29-e70562-s005.docx]

| **Age** | **Gender** | **Metro, Rural Regional** | **language spoken at home** | **Communication Preference and supports** | **Consent – self** | **support at interview** | **Time in minutes** |
| --- | --- | --- | --- | --- | --- | --- | --- |
| 53 | F | large rural town | English | reads and writes | yes | none | 21 |
| 42 | M | large rural town | English | reads and writes | Yes | support worker present | 10 |
| 30 | M | metro | English | reads and writes | yes | no | 13 |
| 36 | M | metro | English | reads with some assistance, not confident to write | yes | researcher assisted with some reading | 13 |
| 28 | M | metro | Cantonese | reads and writes | yes | none | 32 |
| 39 | F | metro | French and English | likes easy read | yes | none | 15 |
| 31 | F | metro | Arabic | writes name, no reading | yes | information read and explained | 13 |
| 38 | M | metro | English | no reading or writing, blind | yes | all questions read out and pressed options for participant - no vision | 12 |
| 42 | M | metro | Cambodian | reads and writes | yes | none | 8 |
| 20s | F | rural town | English | reading - testing online with support | yes | reading and explaining concepts | 25 |
| 58 | F | rural town | English | writes name, no reading | yes | support worker assisted reading out questions | 19 |

S3 : User testing interview participant summary
