# Supplementary material for: Co‐Producing a Patient Reported Experience Measure (PREM) With and for People With Intellectual Disability
Source: Health Expect. 2026 Jan 23;29(1):e70562. doi: 10.1111/hex.70562 (PMC12828785; doi:10.1111/hex.70562)
Supplement: Supplementary file 4 — Listento Me PREM User Guide. [file HEX-29-e70562-s001.pdf]

Listen to Me PREM v 1.0 – Draft instructions for administrators

These instructions are a guide and will be tailored to sites during pilot work.

The Listen to Me PREM is a 9-item measure designed to be completed by people with intellectual disability during or after a healthcare appointment or hospital admission. There is currently no time limit within which the PREM must be completed. The PREM is designed to gather the experiences of people with intellectual disability. Sometimes people may require support to communicate their experiences using the Listen to Me PREM and this is encouraged when required.

Step 1: Determine whether the Listen to Me PREM is suitable for the patient/consumer.

The Listen to Me PREM is designed for people with intellectual disability. Whilst designed or and with people who have intellectual disability, the PREM may also be more accessible for other patients who prefer brief, more interactive, non-text based communication options.

Step 2: Establish whether the person completing the PREM requires support.

Think about the best person to offer support in your setting to enable the person with intellectual disability to explore and record their experiences. This may be a family member, staff or other supporter. Support may include assistance such as accessing the PREM link, understanding the questions or selecting options.

Step 3: Ensure access to the Listen to Me PREM on the preferred device.

The Listen to Me PREM is accessed via a webpage link that is suitable for use with most devices. A device with a touch screen, such as a tablet or laptop, offers greatest functionality for audio and response option selection. If using a computer or laptop without a touch screen, it is possible to click on responses using the mouse. Locate the link, click once and the survey will begin:

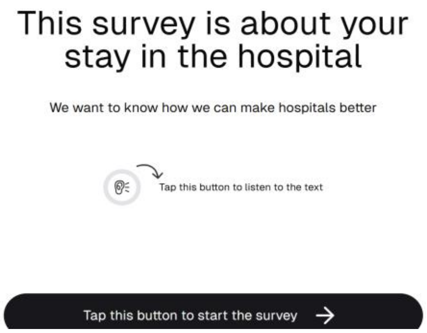

Step 4: Administer the PREM

Individuals can complete the PREM independently or with support as needed at a pace to suit individual needs.

An individual reads or listens to the question using the audible prompt option.

The electronic PREM contains audible prompts, questions can be read out multiple times as needed via the electronic device, or by a supporter if preferred

An audible option is available for all text by pressing the 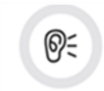 symbol.

The individual selects a response by using the touch screen or mouse click on the desired image.

To move to the next question the large button with arrow at the bottom of the screen is selected.

For questions where text options are requested a keyboard will pop up and can be used to complete.

Step 5: Finishing PREM

When you reach the final screen the following message is displayed.

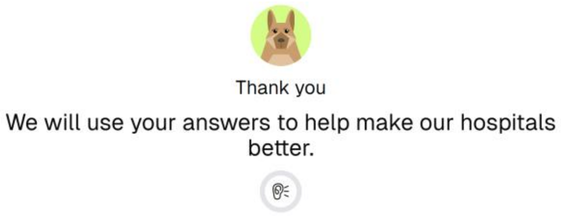

You can click to exit the survey and data collection is complete.

Step 6: Administrator data access and scoring.

Data will be arranged by Macquarie University for each site. Scoring values for closed questions have been included in the PREM development as follows:

Items	Response Options and Scoring		
Questions 1-8	No<br>0	Sometimes<br>5	Yes<br>10

Qualitative free text answers will be included in data set as text.
