# Supplementary material for: Co‐Producing a Patient Reported Experience Measure (PREM) With and for People With Intellectual Disability
Source: Health Expect. 2026 Jan 23;29(1):e70562. doi: 10.1111/hex.70562 (PMC12828785; doi:10.1111/hex.70562)
Supplement: Supplementary file 5 — Listen to Me Hospital Prem Items. [file HEX-29-e70562-s003.pdf]

listentome

This survey is about your stay in the hospital

We want to know how we can make hospitals better

Where did you go to hospital?

Type your location in the box below

Prefer not to say

Section 1 : About You

Do you need help to fill in this survey?

Yes

No

How old are you?

Type your age below

Prefer not to say

What is your gender?

Male

Female

Non-binary

Prefer not to say

Do you have intellectual disability?

Yes

No

Prefer not to say

What language do you speak at home?

Type your languages in the box below

Prefer not to say

English

Section 2: About your time in hospital

Did the people who work at the hospital get to know you?

Yes	Sometimes	No

Did the people who work at the hospital find out how you like to communicate?

Yes	Sometimes	No

Did the people who work at the hospital listen to you?

Yes	Sometimes	No

Did the people who work at the hospital tell you what you need to do?

Yes | Sometimes | No

Did the people who work at the hospital tell you things in a way that you understand?

Yes | Sometimes | No

Did the people who work at the hospital let you ask questions?

Yes | Sometimes | No

Did you feel safe when you were staying in hospital?

Yes

Sometimes

No

Do you think this is a good hospital for people who need care?

Yes

Maybe

No

Is there anything else you would like to tell us?

Type your answer in the box below

Prefer not to say
